# Decreased steroidogenic enzyme activity in benign adrenocortical tumors is more pronounced in bilateral lesions as determined by steroid profiling in LC-MS/MS during ACTH stimulation test

**DOI:** 10.1530/EC-22-0063

**Published:** 2022-06-22

**Authors:** Fidéline Bonnet-Serrano, Maxime Barat, Anna Vaczlavik, Anne Jouinot, Lucas Bouys, Christelle Laguillier-Morizot, Corinne Zientek, Catherine Simonneau, Etienne Larger, Laurence Guignat, Lionel Groussin, Guillaume Assié, Jean Guibourdenche, Ioannis Nicolis, Marie-Claude Menet, Jérôme Bertherat

**Affiliations:** 1 Université Paris Cité, Paris, France; 2 Inserm U1016-CNRS UMR8104, Paris, France; 3 Hormonology Department, Cochin Hospital, Paris, France; 4 Radiology Department, Cochin Hospital, Paris, France; 5 Reference Center for Rare Adrenal Diseases, Endocrinology Department, Cochin Hospital, Paris, France; 6 INSERM, Physiopathologie et Pharmacotoxicologie Placentaire Humaine : Microbiote Pré & Post natal, Paris, France; 7 Diabetology Department, Cochin Hospital, Paris, France; 8 UR 7537 BioSTM, Paris, France; 9 Institut de Chimie Physique, Université Paris-Saclay-CNRS, UMR8000, Orsay, France

**Keywords:** adrenocortical tumors, steroids, LC-MS/MS, ACTH, primary bilateral macronodular adrenal hyperplasia

## Abstract

**Objective:**

Large response of steroid precursors, including 17-hydroxyprogesterone, to adrenocorticotropic hormone (ACTH) has been described in adrenocortical tumors, suggesting the existence of intra-tumoral enzymatic deficiencies. This study aimed to compare steroidogenesis enzymes activity in unilateral and bilateral benign tumors using serum steroid profiling in liquid chromatography coupled with tandem mass spectrometry (LC-MS/MS) in the basal state and after ACTH 1-24 stimulation.

**Design and methods:**

A serum profile of seven consecutive adrenal steroids was determined in LC-MS/MS in the basal state (T0) and after ACTH 1-24 stimulation (T60) in 35 patients with bilateral adrenocortical tumors (BL), 38 patients with unilateral tumors (UL) and 37 control subjects (CT). Response amplitude of each individual steroid was evaluated by T60/T0 ratio, whereas enzymatic activity was assessed by the downstream/upstream steroid ratio. Adrenal volume was quantified by a semi-automatic segmentation method.

**Results:**

For the seven steroids assayed, the amplitude of response to ACTH was higher in BL than in UL and in CT. The difference between BL and UL persisted even after matching patients on adrenal volume. On glucocorticoids pathway, enzymatic activity of CYP11B1 was significantly decreased in BL (78.3 (43.1-199.4)) in comparison to both UL (122.7 (13.8-228.4), *P* = 0.0002) and CT (186.8 (42.1-1236.3), *P* < 0.0001). On mineralocorticoids and androgens pathways, the enzymatic activity of CYP11B2 and CYP17A1-17,20 lyase was also lower in BL than UL and CT.

**Conclusions:**

Decreased activity of distal steroidogenesis enzymes CYP11B1, CYP11B2 and CYP17A1-17,20 lyase, responsible for an explosive response to ACTH of upstream precursors in bilateral tumors, limits the synthesis of bioactive steroids, in particular cortisol, despite the increase in adrenal mass.

**Significance statement:**

Activity of distal steroidogenesis enzymes (CYP11B1, CYP11B2 and CYP17A1 on glucocorticoids, mineralocorticoids and androgens pathways, respectively) is decreased in adrenocortical benign tumors. This decrease is more pronounced in bilateral lesions and seems to depend more on the nature of the lesion than on the increase in adrenal volume. It is responsible for the explosive response to ACTH of steroid precursors located upstream of these enzymes. It probably allows bioactive steroids, particularly cortisol, to stay in the normal range for a long time despite the increase in adrenal mass.

## Introduction

Adrenal incidentalomas, corresponding to clinically inapparent adrenal masses, fortuitously discovered on imaging, are frequent in the general population with a reported prevalence, increasing with age, ranging from 0.5 to 7% (1, 2, 3, 4). About 80% (33–96% among studies) of these adrenal incidentalomas are benign adrenocortical adenomas (5), 7.8–15% of them being bilateral (6). Adrenocortical adenomas are most often non-functioning in 75% of cases (71–84% among series) but they can be responsible for autonomous cortisol secretion in about 12% of cases (1–29% among series) and for aldosterone secretion in 2.5% of cases (1.6–3.3% among series) (5).

Primary bilateral macronodular adrenal hyperplasia (PBMAH) is a form of bilateral benign adrenocortical tumors, responsible for hypercortisolism of progressive installation, often diagnosed between 40 and 65 years and characterized by the presence of bilateral macronodules (diameter >1 cm) on imaging. Differential diagnosis with simple bilateral incidentalomas is not always easy, particularly at the initial stages of disease in the presence of one or two isolated macronodules in each adrenal. In this context, only the evolution of disease will allow a proper diagnosis. In PBMAH, hypercortisolism is a consequence of a global steroidogenesis dysregulation, as suggested by the early elevation of urinary 17-hydroxycorticosteroids (7), relying on two main mechanisms. The first one is the expression of illegitimate G-protein coupled membrane receptors, responsible for a cortisol response to non-physiological stimuli (8, 9, 10). The second one is the secretion of an ectopic intra-adrenal adrenocorticotropic hormone (ACTH) by clusters of adrenocortical cells located in the subcapsular region and in adrenocortical nodules, probably responsible for a paracrine stimulation of cortisol secretion by neighboring cells (11). The underlying mechanisms of these two alterations have not been fully elucidated yet. Indeed, the most common genetic alterations in PBMAH are germline inactivating mutations of the tumor suppressor gene armadillo repeat containing 5 (*ARMC5*) (12), found in 21–26% of PBMAH cases (13, 14). *ARMC5* mutations are associated with a more severe phenotype in terms of both adrenal mass and cortisol secretion level (12, 14). However, no link has been established yet between ARMC5 inactivation and illegitimate receptors expression or intra-adrenal ectopic ACTH secretion. More recently, germline-inactivating mutations of *KDM1A* coding for an histone demethylase have been identified in PBMAH patients presenting food-dependent Cushing syndrome associated with GIP receptor ectopic expression (15, 16, 17). ACTH1-24 stimulation test is not only used to make the diagnosis of adrenal insufficiency, characterized by an insufficient response of cortisol, but is also very useful to detect partial enzymatic deficiency, characterized by an explosive response of the steroid precursor, located upstream of the deficient enzyme. In this context, CYP21A2 enzyme is the most frequently affected in patients with congenital adrenal hyperplasia (CAH), leading to an excessive response of 17-hydroxyprogesterone (17OHP) to ACTH1-24. In incidentally found bilateral adrenocortical (BL) tumors, the ACTH1-24 stimulation test is systematically performed to rule out the diagnosis of CAH, in relation to partial enzymatic deficiency. However, exaggerated response of 17OHP to ACTH has also previously been reported in both UL and BL adrenocortical incidentalomas out of any context of CAH. This has led to the emergence of the concept of intra-tumoral CYP21A2 enzyme deficiency, corresponding to a decreased enzymatic activity in adrenocortical tumoral cells (18, 19, 20).

This study aims to explore steroidogenesis responsiveness to ACTH and to compare steroidogenesis enzymes activity in UL and BL adrenocortical benign tumors using serum steroid profiling in liquid chromatography coupled with tandem mass spectrometry (LC-MS/MS) in the basal state and after ACTH 1-24 stimulation.

## Materials and methods

### Patients and samples

Patients investigated in the Endocrinology department of Cochin Hospital between 2018 December and 2021 April for UL or BL benign adrenocortical tumors and having a Synacthen® (ACTH 1-24) test during hospitalization in this context were included in our study. CAH, primary hyperaldosteronism and the recent use of exogenous glucocorticoids represented exclusion criteria, leading to the inclusion of 38 UL and 35 BL patients. Among the 35 BL patients, 8 patients presented bilateral incidentalomas (ABL), based on the presence of only one nodule on each adrenal, one or both of them being <1 cm of diameter and 27 patients were diagnosed with primary bilateral adrenocortical hyperplasia (PBMAH) defined by the presence of one or more macronodules >1 cm of diameter on each adrenal. Among this subgroup of PBMAH, an *ARMC5* germline mutation was identified in 7 patients. Thirty-seven patients hospitalized in Cochin Hospital Endocrinology or Diabetology departments during the same period, investigated with a Synacthen® stimulation test, outside a context of adrenocortical tumor, and without any abnormality of the corticotroph axis, were also included and considered as controls (CT). Synacthen® test indication in this subgroup was a suspicion of either pituitary disease or CAH or adrenal insufficiency, all these diagnoses having been ruled out. All CT subjects presented a normal basal cortisol level and a normal response of cortisol to Synacthen® test.

Synacthen® test consisted of two blood samples, before (T0 at 08:00 h) and 60 min (T60 at 09:00 h) after 0.25 mg tetracosactide (ACTH1-24, Synacthen®) injection, addressed to the hormonology department. After a 10-min centrifugation at 1800 ***g*** at 4°C, the serum was collected and stored at −20°C before adrenal steroid determination by LC-MS/MS.

Clinical and routine biological data of all included patients were collected from Cochin electronic medical record: demographic characteristics (gender and age); adrenocortical tumor diagnosis; cortisol secretion data (urinary free cortisol and serum cortisol after overnight 1 mg dexamethasone suppression test); complications of a potential Cushing syndrome (weight, BMI, high blood pressure, diabetes, dyslipidemia and osteoporosis).

The project was approved by the Ethical Review Committee for publications of the Cochin University Hospital (CLEP) on December 14, 2020, as a non-interventional retrospective monocentric analysis, all patients included in the analysis gave their informed consent for the collection of data as part of their medical care. For BL and UL patients, an additional informed written consent was obtained for the study including genetic analysis with approval from the Ile de France I Ethic committee.

### Mass spectrometry analysis

**Figure 1:**
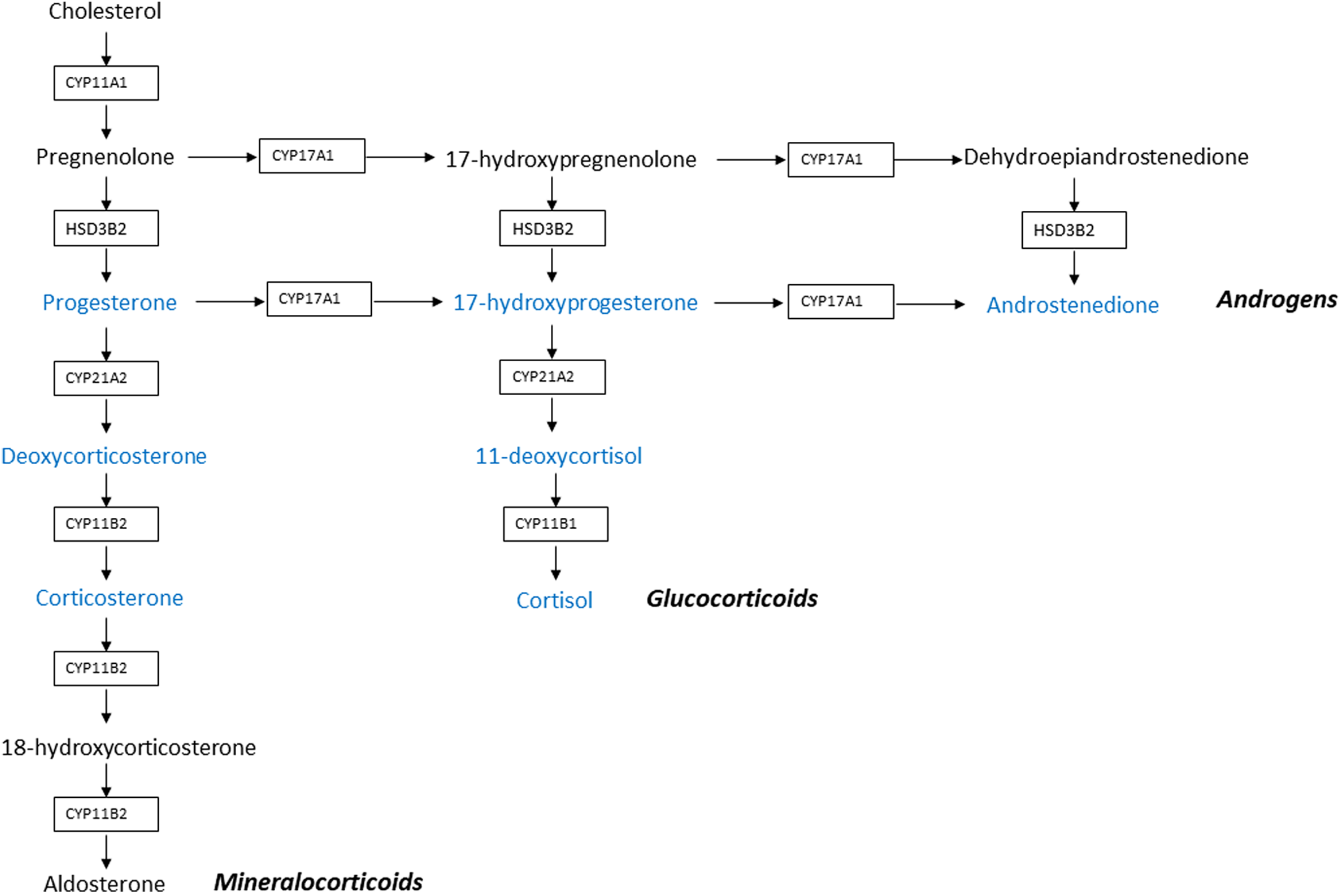
Adrenal steroidogenesis diagram. Steroidogenic enzymes are represented in the boxes. The three steroidogenesis pathways are indicated in bold italics. The seven molecules belonging to the analyzed steroid profile are given in blue.

A profile of seven steroids of interest, that is, directly produced by adrenals (not resulting from peripheric conversion), and consecutive along the three different pathways (glucocorticoids, androgens and mineralocorticoids), was determined using LC-MS/MS. They are shown in blue in Fig. 1.

### Reagents

Ethyl ether and HPLC-grade water were supplied by CARLO ERBA (Italy). HPLC-grade methanol (MeOH) was purchased from Thermo Fischer Scientific.

6 PLUS1 Multilevel Serum Calibrator Set MassChrom Steroids Panel 1&2 allowing the simultaneous quantification of 7 steroids (progesterone, 11-deoxycorticosterone, corticosterone, 17OHP, 11-deoxycortisol, cortisol and androstenedione) among the proposed panel of 15 steroids was purchased from Chromsystems (Germany), as well as internal quality controls (QC) MassCheck Steroid Panel 1&2 Serum Control.

Internal standards: corticosterone-D8 and 11-deoxycorticosterone-D8 were purchased from LGC (Teddington, UK) and separately dissolved in MeOH to get stock solutions at 1 mg/mL, stored at −20°C. Internal standard solutions of cortisol-D4, 11-deoxycortisol-D5, androstenedione-^13^C3, 17OHP-D8 and progesterone-D9 were purchased from Sigma-Aldrich. A mix of the 7 internal standard solutions was further prepared in MeOH.

### Steroids extraction from serum

One hundred microliters of serum (or standard or QC) were vortex-mixed with 30 µL of the 7 internal standards mix solution and 1.5 mL of ethyl ether for 3 times for 1 min. After 10 min of rest, samples were then frozen at −80° for 8 min. The ether supernatants were separated and evaporated to dryness at 37°C under a steam of nitrogen. Dried extracts were further reconstituted in 100 µL of MeOH/water (50/50).

### Conditions and instrumentation for ultra-high-performance liquid chromatography-mass spectrometry

Steroid profiles were characterized using LC-MS/MS. Ten microliters of each sample were injected in a TSQ-ALTIS instrument from Thermo Fischer equipped with an Acquity UPLC HSS T3 column (2.1 mm of internal diameter, 50 mm of length) which was purchased from Waters (Milford, MA, USA). Mobile phase was composed of a mix of MeOH and water with 0.1% of ammonium acetate and 0.1% of formic acid. It was operated at a flow rate of 0.513 mL/min at a temperature of 40°C for a run of 11 min. Molecules at the output of UPLC column were ionized using positive ElectroSpray Ionisation and mass spectra analysis was made in multiple reactions monitoring data acquisition mode. Transitions used for the quantification and confirmation of each steroid and corresponding internal standard as well as their expected retention time are reported in Supplementary Table 1 (see section on supplementary materials given at the end of this article). Data processing was made using Thermo Fischer software Tracefinder 4.0 Quan.

### Adrenal imaging analysis

Adrenal computed tomography exams available for UL and BL patients were retrospectively analyzed by a single investigator (MB) to confirm the benign nature of the lesions based on the consensus statement of the European Society of Endocrinology on the management of adrenal incidentalomas (5) (including spontaneous density <10 HU, relative washout >40% and absolute washout >60%) and to evaluate global adrenal volume as well as main adrenal nodule volume via a semi-automatic segmentation method. CT scans were exported from the picture archiving and communication system (PACS) in the digital imaging in communications and medicine format and imported in the 3DSlicer® software (21). The whole adrenal gland tissue was semi-automatically segmented in three dimensions using the ITK SNAP 2.0.2 included in the 3DSlicer® software 4.13.0 (21). In patients with a dominant nodule, this one was segmented with the whole gland and individualized. The volume was calculated using the shape voxel volume after normalization of voxels using 1 × 1 × 1mm shape with 1 voxel = 1 µL.

### Statistical analysis

All statistical analyses were performed using the R software (https://www.r-project.org/). The comparison between consecutive steroids along steroidogenesis pathways was made using paired Wilcoxon tests. The comparison between the three groups of subjects (CT, UL and BL) was performed using Dunn’s test (https://CRAN.R-project.org/package=dunn.test) after Kruskal–Wallis test. False discovery rate was controlled by applying the Benjamini–Hochberg *P* - value adjustment for multiple comparisons (22). The same tests were used to compare the three groups of subjects based on cortisol level secretion (normal N, subclinical SC and excessive CS cortisol secretion) and between the three groups of bilateral tumors (bilateral adenomas, WT *ARMC5* PBMAH and mutated *ARMC5* PBMAH). The comparison between *ARMC5* mutated and *ARMC5* WT bilateral tumors was performed using the Wilcoxon test. The comparison between UL and BL patients after matching on adrenal volume (difference <15%) was performed using the Wilcoxon matched-pairs signed-rank test. The results were expressed as median (minimum-maximum). For all box and whisker plots, the black line inside the rectangle represented median values. First (Q1) and third (Q3) quartiles corresponded to the lower and upper border of the rectangle, respectively. Whiskers low and up extremities were calculated by the respective formula: max (min; Q1 – 1.5 x (Q3-Q1)) and min (max; Q3 + 1.5 x (Q3-Q1)). Dots represented extreme values outside the whiskers.

## Results

## Characteristics of study subjects

### Clinical characteristics

The investigated population included most of women (74%). Sex ratio (SR) (F/M) was not significantly different between control subjects (4.3) and patients with unilateral (SR = 2.4) or bilateral (SR = 2.2) adrenal tumors (*P* = 0.776) (Table 1). Control subjects were younger (38 (16–89) years) than patients with unilateral (63.5 (31–85) years; *P* = 0.0001) and bilateral tumors (62 (36–77) years; *P* = 0.0002). Control subjects had also a lower BMI (23 (18–40.4) kg/m^2^) than both UL (25.4 (16.6–44.6) kg/m^2^; *P* = 0.0227) and BL patients (28 (19.2–42.1) kg/m^2^; *P* <0.0001). No significant difference was found between UL and BL patients either on age or BMI (*P* = 0.719and *P* *= *0.255, respectively).

**Table 1:** Clinical characteristics, routine biological results and radiologic data in the three groups: CT subjects, UL and BL patients.

	CT subjects	UL patients	BL patients	*P* value, UL vs BL patients
Number of subjects	37	38	35	
Clinical characteristics				
Age (years)	38 (16–89)	63.5 (31–85)	62 (36–77)	0.719
Gender	30F/7M	27F/11M	24F/11M	0.776
BMI (kg/m^2^)	23 (18–40.4)	25.5 (16.6–44.6)	28 (19.2–42.1)	0.255
Routine biological data				
Cortisol after dexamethasone minute suppression test (nM)	28 (16–29)	54 (19–535)	68 (20–680)	0.246
Free urinary cortisol (folds change/*N*)	0.43 (0.2–0.62)	0.54 (0.11–2.54)	0.45 (0.14–7.25)	0.885
ACTH (pmol/L)	5.1 (2–15.4)	3.8 (0.4–20.5)	2.3 (0.4–8.1)	0.00413
Overt Cushing (%)	0%	18%	19%	
Subclinical Cushing (%)	0%	35%	44%	
Radiologic data				
Bilateral adrenal volume		13,524 (5385–106,170)	20,449 (8151–157,915)	0.0175

### Radiological characteristics

As expected, the total adrenal volume was significantly higher (20,449 (8151–157,915) µL) in BL patients than in UL patients (13,524 (5385–106,170) µL; *P* = 0.0175) with a large inter-individual variability inside each group of tumors.

### Routine hormonal results

Serum cortisol after 1 mg dexamethasone suppression test as well as free urinary cortisol were not significantly different between UL patients and BL patients (54 (19–535) nmol/L vs 68 (20–680) nmol/L; *P* = 0.246and 0.54 (0.11–2.54) vs 0.45 (0.14–7.25) times upper limit of normal; *P* *= *0.885, respectively). Among UL patients, 6/34 (18%) and 12/34 (35%) had a serum cortisol after 1 mg dexamethasone suppression test superior to 138 nmol/L and between 50 and 138 nmol/L, respectively. Among BL patients, 6/32 (19%) and 14/32 (44%) had a serum cortisol after 1 mg dexamethasone suppression test superior to 138 nmol/L and between 50 and 138 nmol/L, respectively. ACTH value was significantly lower in BL patients (2.3 (0.4–8.1) pmol/L) in comparison to UL patients (3.8 (0.4–20.5) pmol/L, *P* = 0.0041).

### Differences in serum steroid profiles at the basal state and after ACTH1-24 stimulation between the three groups

**Figure 2:**
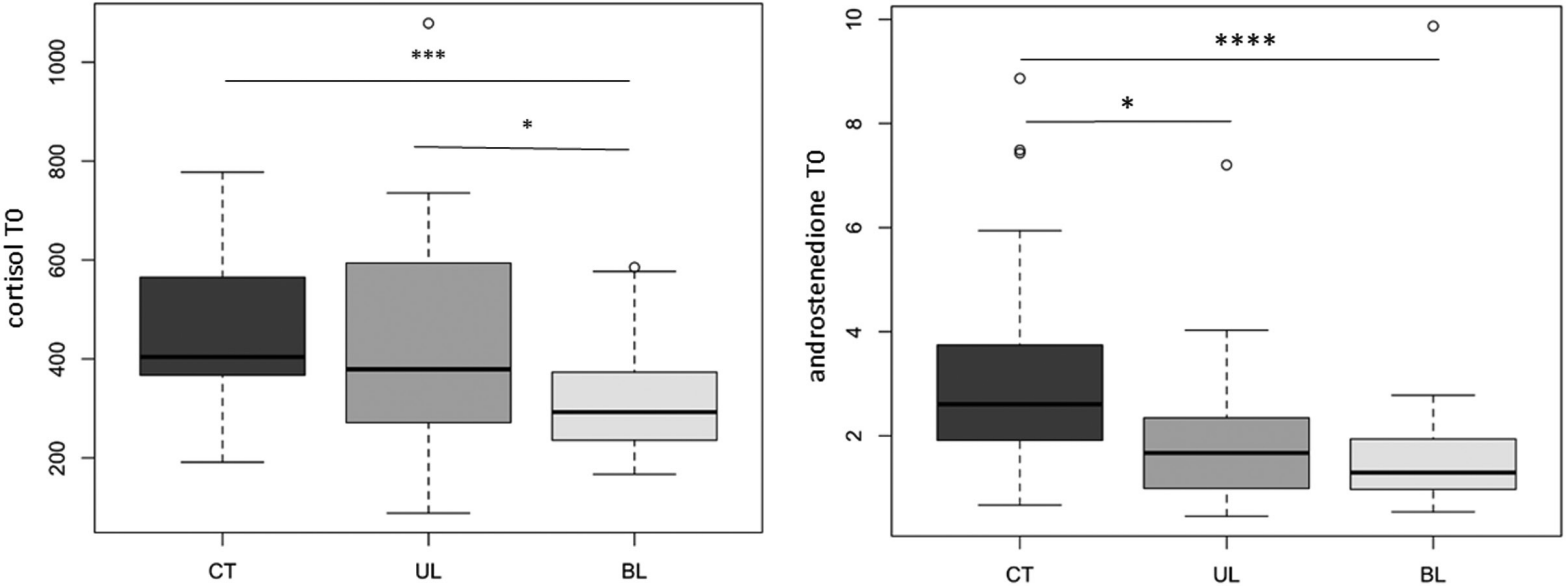
Comparison of cortisol and androstenedione levels at basal state (T0) between CT subjects, UL patients and BL patients. Cortisol basal level is lower in BL patients in comparison to both UL patients and CT subjects. Androstenedione basal level is lower in UL and BL patients in comparison to CT subjects. Results are expressed in nmol/L. *P <  0.05 ; ***P <  0.0001; ****P < 0.00001.

At the basal state (T0), cortisol level was unexpectedly lower in BL patients (309.8 (167.2–585.2) nmol/L) than in UL patients (379.2 (88.5–1078.6) nmol/L; *P* = 0.0317) and in CT subjects (404.1 (191.6–777.8) nmol/L; *P* = 0.0036) (Fig. 2). Androstenedione level was also lower in UL (1.67 (0.45–7.2) nmol/L) and BL patients (1.25 (0.54–2.78) nmol/L) in comparison to CT subjects (2.61 (0.67–8.87); *P* = 0.00194 and *P* < 0.0001, respectively) (Fig. 2). No significant difference was observed for the five other steroids at basal state (Supplementary Fig. 1).

After ACTH1-24 stimulation (T60), no significant difference was found for either cortisol or androstenedione but the level of the five other steroids was higher in BL patients in comparison to both UL patients and CT subjects (Supplementary Fig. 2).

Study subjects were classified into three groups according to their cortisol level after 1 mg dexamethasone suppression test when available (6/37 CT subjects, 34/38 UL patients and 32/35 BL patients) in normal (N, *n*  =33) corresponding to cortisol level < 50 nmol/L, subclinical (SC, *n* = 27) corresponding to cortisol level between 50 and 138 nmol/L and excessive cortisol secretion (CS, *n* = 12) corresponding to cortisol level > 138 nmol/L, respectively. At the basal state (T0), corticosterone level was higher in SC patients than in N patients (*P* = 0.0149) and 11-deoxycortisol was higher in SC and CS than in N patients (*P* = 0.0167 and *P* = 0.0264, respectively). No difference was found for either cortisol, androstenedione, deoxycorticosterone, 17OHP or progesterone (Supplementary Fig. 5). After ACTH stimulation (T60), the only significant difference observed was for 11-deoxycortisol higher in CS than in N patients (*P* = 0.0202). (Supplementary Fig. 6).

### Higher amplitude of response to ACTH for all steroids in BL patients in comparison to both UL patients and CT subjects

For all studied steroids, the amplitude of response to ACTH was evaluated by the ratio of concentrations T60/T0 (i.e. concentration after ACTH1-24 stimulation at the numerator and concentration at basal state at the denominator).

**Figure 3:**
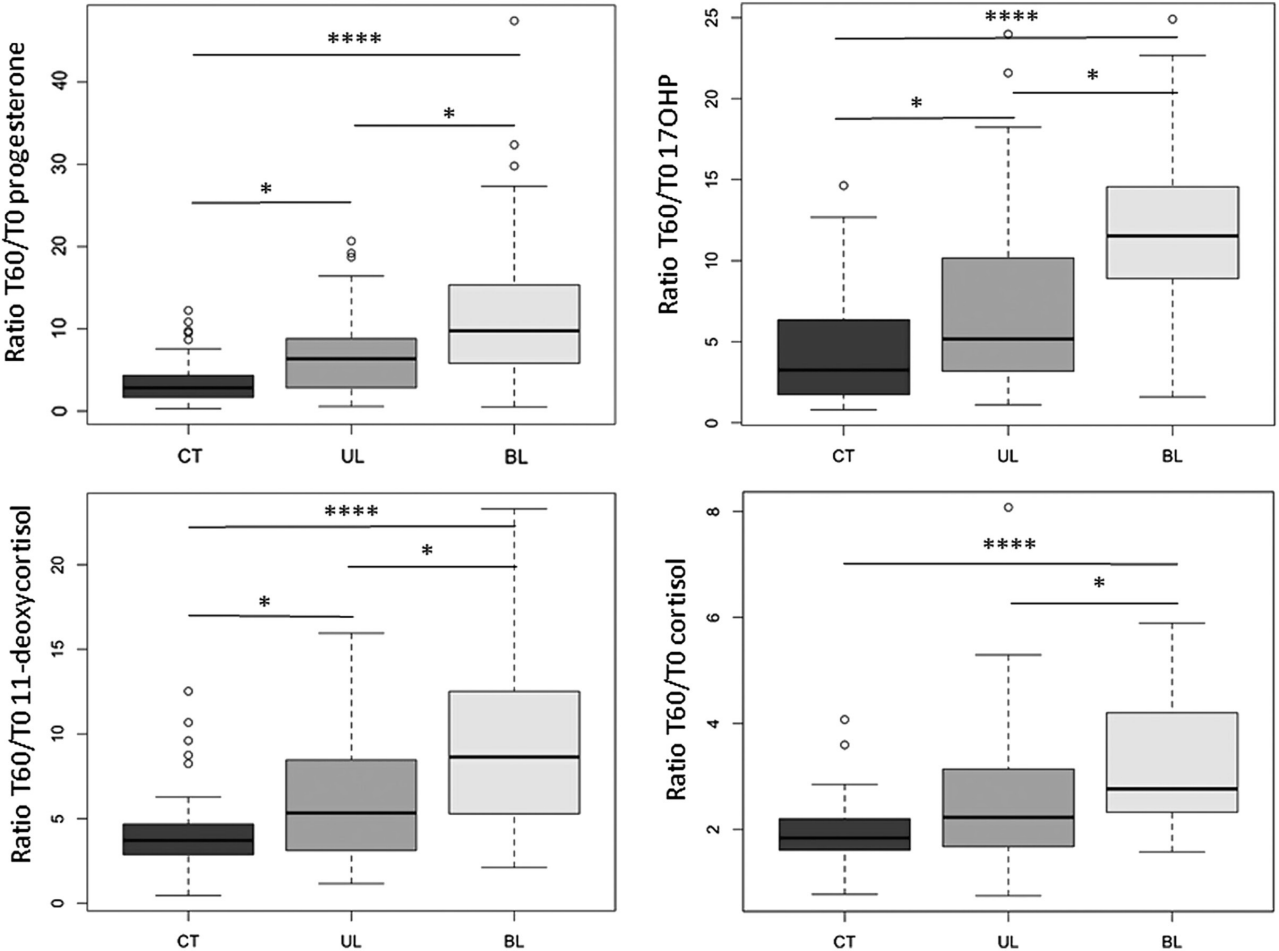
Comparison of the amplitude of response to ACTH of glucocorticoids and precursors between CT subjects, UL patients and BL patients. The amplitude of response is higher in BL patients in comparison to both CT subjects and UL patients. 17OHP: 17-hydroxyprogesterone.* *P* <  0.05; ** *P* < 0.001; **** *P* < 0.00001.

Along the glucocorticoids pathway, the amplitude of response to ACTH of the three consecutive precursors (progesterone > 17OHP > 11-deoxycortisol) and of the bioactive cortisol molecule was significantly higher in BL patients in comparison to both CT subjects and UL patients. Thus, T60/T0 progesterone ratio was higher in BL patients (9.8 (0.5–47.4)) than in UL patients (6.4 (0.6–20.7), *P* = 0.0054) and CT subjects (2.8 (0.3–12.2), *P* < 0.0001). T60/T0 17OHP ratio was also higher in BL patients (11.5 (1.6–24.9)) than in UL patients (5.2 (1.1–24.0), *P* = 0.0030) and CT subjects (3.3 (0.8–14.6), *P* <0.0001) and T60/T0 11-deoxycortisol ratio was higher in BL patients (8.6 (2.1–23.3)) than in UL patients (5.3 (1.2–15.9), *P* = 0.0070) and in CT subjects (3.7 (0.5–12.5), *P* < 0.0001). Finally, T60/T0 cortisol ratio was higher in BL patients (2.8 (1.6–5.9)) than in UL patients (2.2 (0.8–8.1), *P* = 0.0046) and CT subjects (1.8 (0.8–4.1), *P* < 0.0001) (Fig. 3).

It is also interesting to notice that along glucocorticoids pathway, the amplitude of response to ACTH of cortisol was significantly lower than the amplitude of response of the three upstream precursors (progesterone > 17OHP > 11-deoxycortisol) in the three groups (Supplementary Fig. 3), suggesting a physiological tight regulation of bioactive cortisol amplitude of response to ACTH.

The same differences were also observed along androgens and mineralocorticoids pathways, amplitude of response to ACTH of androstenedione, deoxycorticosterone and corticosterone being also higher in BL patients in comparison to both CT subjects and UL patients (Supplementary Fig. 4).

Interestingly, as far as cortisol secretion level is concerned, no difference was found for any of the seven steroids in terms of amplitude of response to ACTH (T60/T0 ratio), between the three groups N, SC and CS (Supplementary Fig. 7).

### Amplitude of response to ACTH is not correlated with adrenal volume in either BL or UL patients

**Figure 4:**
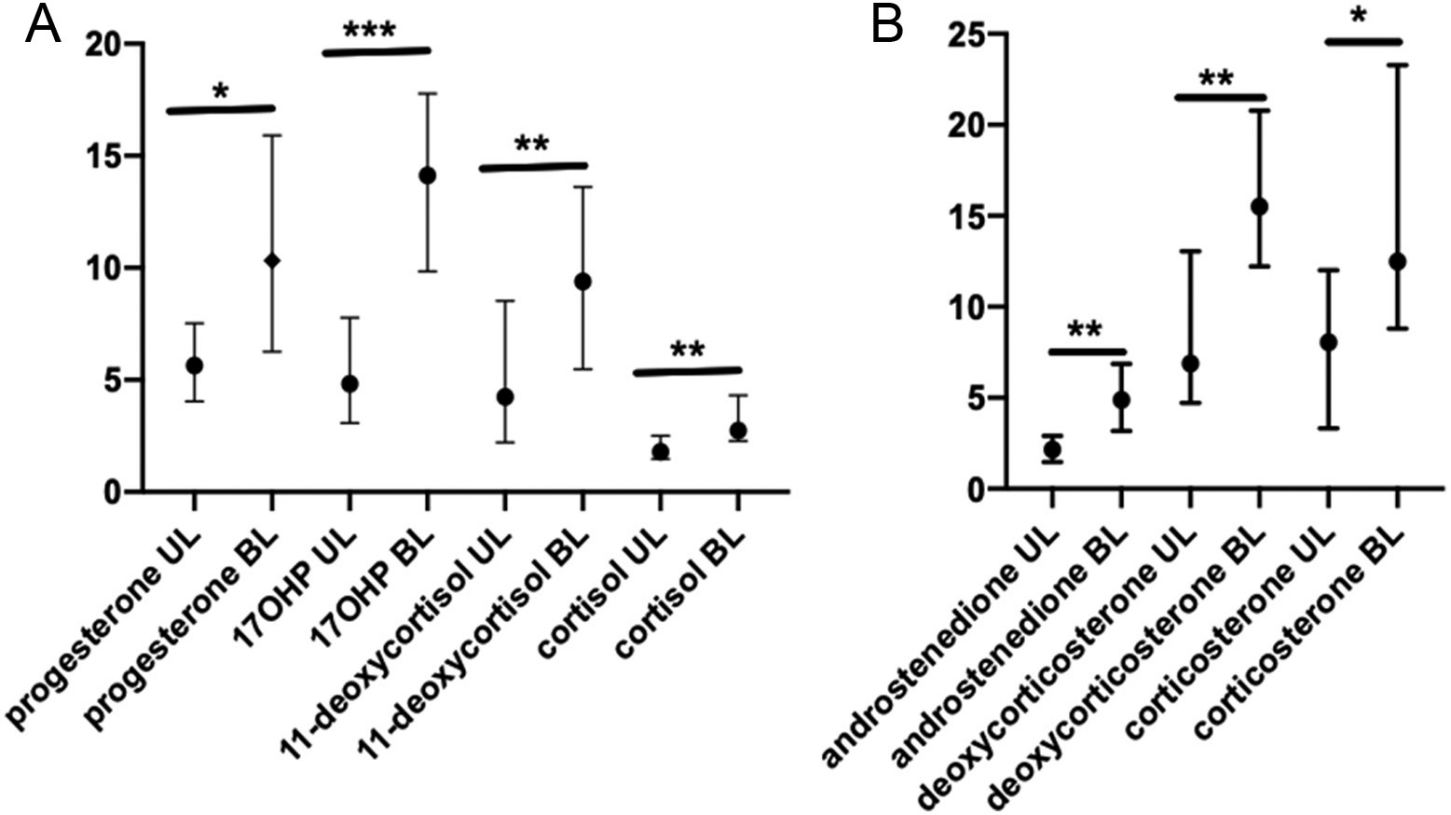
Comparison of steroid response amplitude to ACTH between UL and BL patients after matching on adrenal volume. Steroid amplitude of response to ACTH remains higher in BL patients than in UL patients even after matching on adrenal volume. (A) Glucocorticoids pathway. (B) Other steroids. Results are represented as median and interquartile range. 17OHP: 17-hydroxyprogesterone.*P <  0.05 ; **P < 0.001; ***P <  0.0001.

No correlation was found between the amplitude of response to ACTH of any of the seven steroids studied and adrenal volume in either BL or UL patients. Supplementary Fig. 8 shows the amplitude of response to ACTH of the four consecutive steroids along the glucocorticoids pathway as a function of global adrenal volume in UL (A) and BL (B) patients. Moreover, the difference between BL and UL patients in terms of the amplitude of response to ACTH remained significant for the seven steroids studied, after matching patients on adrenal volume (difference <15%, 17 pairs) (Fig. 4). These observations suggested the existence of other determining factors of steroid amplitude of response to ACTH.

### Decreased enzymatic activity in BL patients in comparison to both UL patients and CT subjects

**Figure 5:**
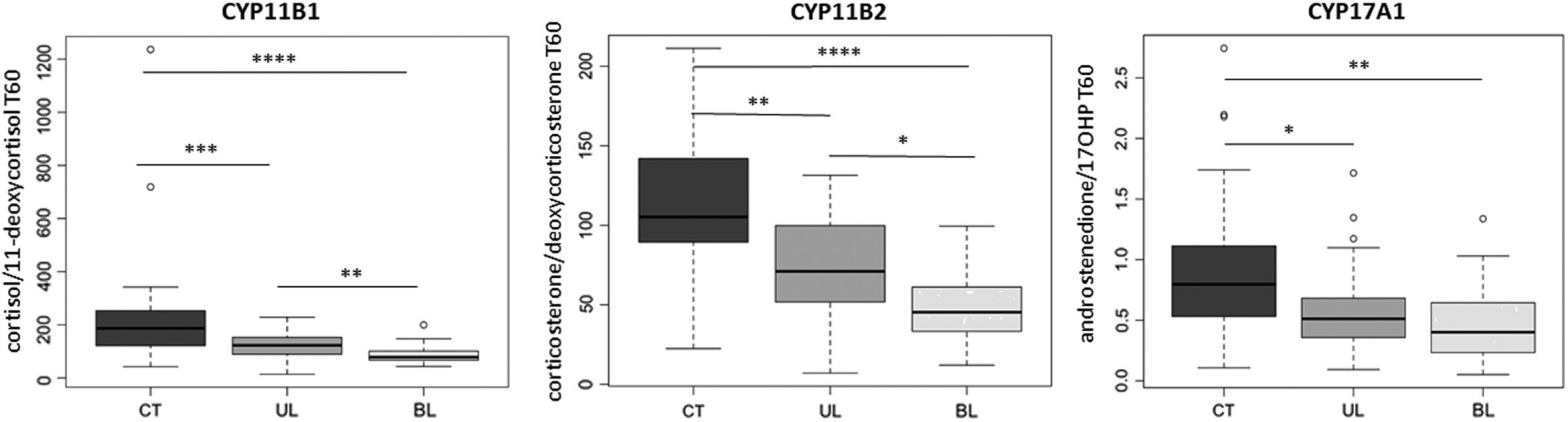
Enzymatic activity of downstream enzymes in CT subjects and UL and BL patients. The enzymatic activity of CYP11B1 (glucocorticoids pathway), CYP11B2 (mineralocorticoids pathway) and CYP17A1-17,20-lyase (androgens pathway) is decreased in BL patients in comparison to both CT subjects and UL tumors. Enzymatic activity was evaluated by downstream/upstream steroid ratio at T60. 17OHP: 17-hydroxyprogesterone.**P < 0.001; ****P < 0.00001.

The activity of adrenal steroidogenesis enzymes was evaluated by the ratio of concentrations of downstream steroid/upstream precursor after ACTH stimulation (T60). Several enzymes showed decreased activity in BL patients in comparison to both UL patients and CT subjects. Thus, CYP11B1 activity, evaluated by T60 cortisol/11-deoxycortisol ratio, was lower in BL patients (78.3 (43.1–199.4)) than in UL patients (122.7 (13.8–228.4), *P* = 0.0002) and CT subjects (186.8 (42.1–1236.3), *P* < 0.0001). Similarly, CYP11B2 activity, evaluated by T60 corticosterone/deoxycorticosterone ratio, was also decreased in BL patients (45.4 (12–99.5)) in comparison to both UL patients (71.1 (7.0–131.4), *P* = 0.0005) and CT subjects (105.3 (22.5–211.3), *P* < 0.0001). Finally, CYP17A1-17,20 lyase, evaluated by T60 androstenedione/17OHP ratio, was also lower in BL tumors (0.4 (0.05–1.34)) than in CT subjects (0.8 (0.11–2.74), *P* < 0.0001) (Fig. 5). No such differences were observed for upstream steroidogenic enzymes including CYP17A1-17alpha-hydroxylase, evaluated by T60 17OHP/progesterone ratio or CYP21A1 evaluated by either T60 deoxycorticosterone/progesterone ratio or T60 11-deoxycortisol/17OHP ratio. Interestingly, the three enzymes whose activity was decreased in BL patients were also significantly less active in BL patients presenting an *ARMC5* mutation (PBMAH Mut) in comparison to BL patients without any *ARMC5* mutation (‘bilateral adenomas’ and ‘WT ARMC PBMAH’ merged into one group under the generic abbreviation ‘BL WT’), the difference being statistically significant for CYP11B1 (*P* = 0.0357*)* and CYP17A1-17-alpha-hydroxylase (*P* = 0.0357*)* (Supplementary Fig. 9).

## Discussion

The first original result of this study is that the seven steroids studied, distributed on the three adrenal steroidogenesis pathways (glucocorticoids, androgens and mineralocorticoids), presented a higher amplitude of response to ACTH in patients with bilateral tumors (BL) not only in comparison to control subjects (CT) but also in comparison to patients with UL tumors. The only previous study comparing steroid profiles after ACTH stimulation between control subjects, UL and BL adrenocortical tumors (23) only looked at basal (T0) and stimulated (T60) steroid levels in absolute value and did not describe steroid amplitude of response to ACTH. Besides, patients’ recruitment was rather different: bilateral tumors only consisted in bilateral incidentalomas (no case of PBMAH reported).

This study presents some limitations. First, it is based on a retrospective monocentric analysis. However, the period of patients’ inclusion was rather short and clinical practices were thus quite homogenous all along the study. Second, bilateral lesions were merged in only one group when they can result from processes of different natures, definitive diagnosis being only made on histology. However, no surgical indication was retained for most of the included patients which made further classification difficult. The last limitation was the composition of the group of CT subjects. Indeed, CT subjects were globally younger than UL and BL patients and they did not systematically perform an adrenal imaging.

Our first hypothesis to explain the higher amplitude of steroid response to ACTH in BL patients was a potential relation with adrenal global volume. Indeed, a correlation between the peak of 17OHP response to ACTH and tumor main diameter in adrenocortical incidentalomas had previously been described (18), suggesting a link between steroid responsiveness to ACTH and adrenal mass. In our study, adrenal volume was quantified far more precisely by a semi-automatic three-dimensional segmentation method in both UL and BL patients. Adrenal volume was, as expected, significantly higher in BL patients: 20,449 (8151–157,915) µL in BL patients than in UL patients 13,524 (5385–106170) µL with a large inter-individual variability in each group. With this approach, no correlation was found between any steroid response amplitude to ACTH and adrenal volume in either BL or UL patients. Moreover, amplitude of response to ACTH of all steroids remained higher in BL patients than in UL patients after matching patients closely on adrenal volume. This observation suggested the existence of other determining factors of steroid responsiveness to ACTH in UL and BL patients.

Steroidogenesis enzymes activities, evaluated by product to substrate ratios, were thus compared between the three groups of subjects. Interestingly, CYP11B1, CYP11B2 and CYP17A1-17,20 lyase, corresponding to the most distal steps of steroidogenesis accessible by our approach on the three different pathways (glucocorticoids, mineralocorticoids and androgens, respectively), showed a decreased activity in BL patients in comparison to both control subjects and UL patients. This was responsible for lower basal level of cortisol and androstenedione in BL patients. On the mineralocorticoids pathway, the same analysis was limited by the absence of aldosterone level determination. These original data update the notion of intra-tumoral enzymatic blocks. Exaggerated response of 17OHP to ACTH had previously been reported in both UL and BL adrenocortical incidentalomas (18, 20, 25), with a higher frequency in BL (67%) than in UL adenomas (50%) (24). This observation was initially attributed to a potential intra-tumoral CYP21A2 deficiency (18, 19, 20, 25). However, all these studies were only based on 17OHP level after ACTH 1–24stimulation test. The few studies (25, 26) describing the response to ACTH 1–24 of several steroids determined by immunoassays reported also a higher progesterone, 17OHP, deoxycorticosterone and 11-deoxycortisol levels in adrenocortical incidentalomas and proposed CYP11B1 and CYP11B2 intra-tumoral deficiency. A more recent study (27) also reported higher 21-deoxycortisol and deoxycorticosterone levels in LC-MSMS after ACTH 1-24 stimulation test in subclinical cortisol-secreting adrenocortical adenomas while basal and stimulated androgens levels (including androstenedione) were decreased. In our study, despite an increase in 17OHP amplitude of response to ACTH 1-24, no decrease in CYP21A2 activity based on either deoxycorticosterone/progesterone or 11-deoxycortisol/17OHP ratios was found. The increase in upstream precursors (i.e, progesterone and 17OHP) response amplitude in BL tumors was thus rather attributed to a decreased activity of enzymes acting downstream CYP21A2. Thus, the decrease in downstream steroidogenesis enzymes (CYP11B1, CYP11B2 and CYP17A1-17,20 lyase) activity in BL patients could explain the increased amplitude of response to ACTH of the five steroid precursors studied: progesterone, 17OHP, 11-deoxycorticosterone, corticosterone and 11-deoxycortisol.

Interestingly, CYP11B1, CYP11B2 and CYP17A1 activities were also decreased in *ARMC5*-mutated PBMAH patients in comparison to other bilateral tumors (BLWT), suggesting an exacerbation of the enzymatic phenotype existing in all BL tumors. This decreased enzymatic activity is concordant with the less effective steroidogenesis previously described in *in vitro* models of *ARMC5* inactivation. Decrease in CYP21A2 and CYP17A1 expression have thus been reported in H295R cell line after *ARMC5* inactivation by siRNA (12). In a more recent study (19) using a model of *ARMC5 s*ilencing in non-mutated PBMAH cells, a decrease in mARN expression of *StAR* *, CYP11A1, CYP17A1, NR5A1*, encoding steroidogenic factor 1 (SF1), and *MC2R*, encoding ACTH receptor, has also been described.

However, this observation does not explain the higher amplitude of response to ACTH of cortisol and androstenedione that we observed in BL patients. The ectopic secretion of intra-adrenal ACTH by clusters of adrenocortical cells, described in PBMAH, could here play a role in this increased amplitude of response. Indeed, adrenocortical cells able to produce ACTH, also express ACTH receptor, MC2R, which suggests a potential amplification of ACTH stimulation, thanks to these specific clusters of adrenocortical cells, via an additional paracrine stimulation of cortisol secretion by neighboring cells (11).

## Conclusion

These observations give interesting perspectives on the understanding of steroidogenesis alterations in adrenocortical bilateral tumors, particularly in PBMAH, characterized by a large increase in adrenal mass, but remain asymptomatic for a long time. This might be explained by a decreased activity of distal steroidogenesis in tumor cells, allowing distal steroids, particularly bioactive cortisol, to stay in the lower limits of the normal range at the basal state. In the situation of acute stimulation by ACTH, this mechanism, based on a decreased activity of distal steroidogenesis enzymes, probably limits but does not completely prevent the higher amplitude of response of distal steroids (cortisol and androstenedione) in BL patients. Moreover, it is also responsible for an explosive response to ACTH of upstream steroid precursors (17OHP and 11-deoxycortisol). In this model, PBMAH could become symptomatic when the decrease in distal steroidogenesis enzymes activity becomes insufficient to compensate for the increase in adrenal mass and to keep basal cortisol in the normal range.

## Supplementary Material

Supplemental Figure 1: Comparison of progesterone, 17-hydroxyprogesterone, 11-deoxycortisol, deoxycorticosterone and corticosterone levels at basal state (T0) between CT subjects, UL patients and BL patients. Results are expressed in nmol/L.

Supplemental Figure 2: Comparison of the different steroids levels after ACTH1-24 stimulation (T60) between CT subjects, UL patients and BL patients. Results are expressed in nmol/L. * p< 0.05 ; ** p<0.001; *** p< 0.0001; **** p<0.00001.

Supplemental Figure 3: Decreasing amplitude of response to ACTH from downstream precursors to downstream bioactive cortisol on glucocorticoid pathways in the 3 groups : CT subjects, UL and BL patients. Amplitude of response to ACTH was evaluated by T60/T0 steroid concentration ratio. 17OHP: 17-hydroxyprogesterone. * p< 0.05 ; ** p<0.001; **** p<0.00001.

Supplemental Figure 4: Comparison of the amplitude of response to ACTH of androstenedione and mineralocorticoids precursors (deoxycorticosterone and corticosterone) between CT subjects, UL patients and BL patients. The amplitude of response is higher in BL patients in comparison to both CT subjects and UL patients. * p< 0.05; *** p< 0.0001; **** p<0.00001.

Supplemental Figure 5: Comparison of progesterone, 17-hydroxyprogesterone, 11-deoxycortisol, cortisol, corticosterone, deoxycorticosterone, and androstenedione levels at basal state (T0) between N patients, SC patients and CS patients (cortisol status level). Results are expressed in nmol/L. * p< 0.05.

Supplemental Figure 6: Comparison of progesterone, 17-hydroxyprogesterone, 11-deoxycortisol, cortisol, corticosterone, deoxycorticosterone, and androstenedione levels after ACTH1-24 stimulation (T60) between N patients, SC patients and CS patients (cortisol status level). Results are expressed in nmol/L. * p< 0.05.

Supplemental Figure 7: Comparison of the amplitude of response to ACTH of progesterone, 17-hydroxyprogesterone, 11-deoxycortisol, cortisol, corticosterone, deoxycorticosterone, and androstenedione between N patients, SC patients and CS patients (cortisol level status).

Supplemental Figure 8: Relation between steroid amplitude of response to ACTH of glucocorticoids and precursors and adrenal volume. No correlation between any steroid amplitude of response to ACTH and adrenal volume was found in either UL (A) or BL patients (B).

Supplemental Figure 9: Enzymatic activity of downstream enzymes in BL patients according to the ARMC5 Genotype. CYP11B1 (glucocorticoids pathway), CYP11B2 (mineralocorticoids pathway) and CYP17A1 (androgens pathway) is decreased in PBMAH with ARMC5 mutation (PBMAH Mut) in comparison to BL patients with no ARMC5 mutation (BLWT). Enzymatic activity was evaluated by downstream/upstream steroid ratio at T60. 17OHP: 17-hydroxyprogesterone.* p< 0.05.

Supplemental Table 1: Quantification and confirmation transitions, and retention time of each steroid molecule.

## Declaration of interest

All the authors have nothing to disclose. Jérôme Bertherat is on the editorial board of *Endocrine Connections*. Jérôme Bertherat was not involved in the review or editorial process for this paper, on which he/she is listed as an author.

## Funding

This study was funded by Agence Nationale de la Recherche http://dx.doi.org/10.13039/501100001665 (18-CE14-0008-01).
