# Supplementary figures and images for: Decreased steroidogenic enzyme activity in benign adrenocortical tumors is more pronounced in bilateral lesions as determined by steroid profiling in LC-MS/MS during ACTH stimulation test
Source: Endocr Connect. 2022 Jun 22;11(8):e220063. doi: 10.1530/EC-22-0063 (PMC9346343; doi:10.1530/EC-22-0063)

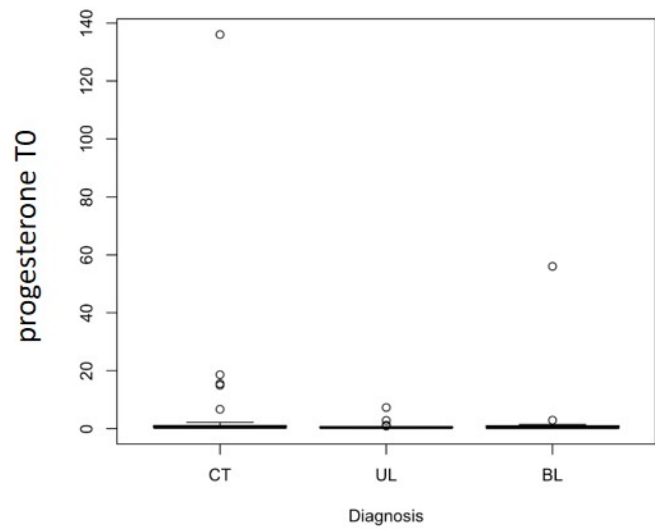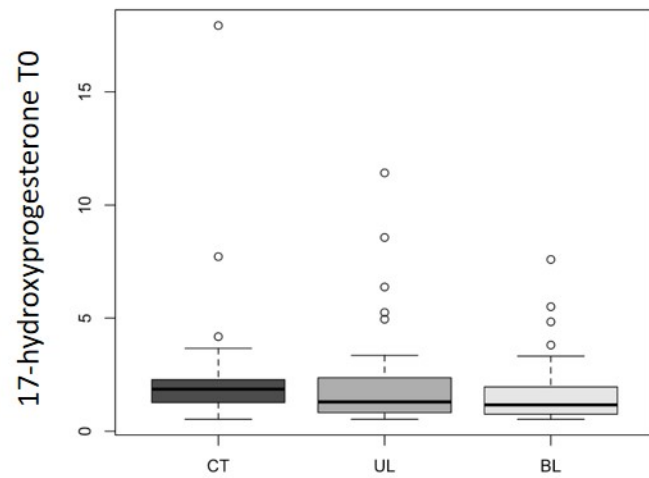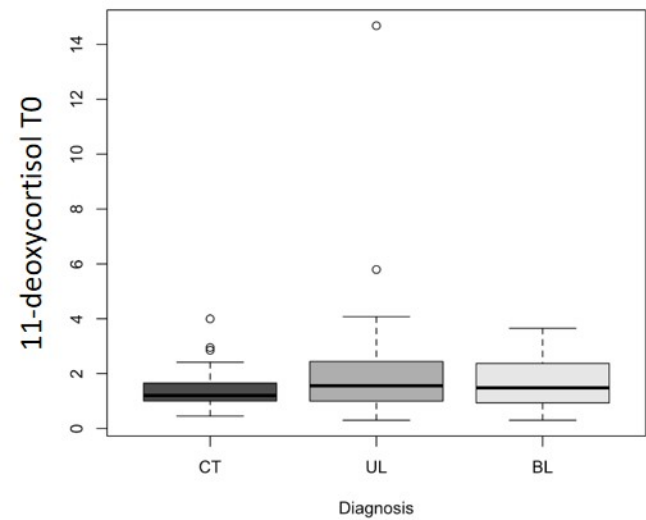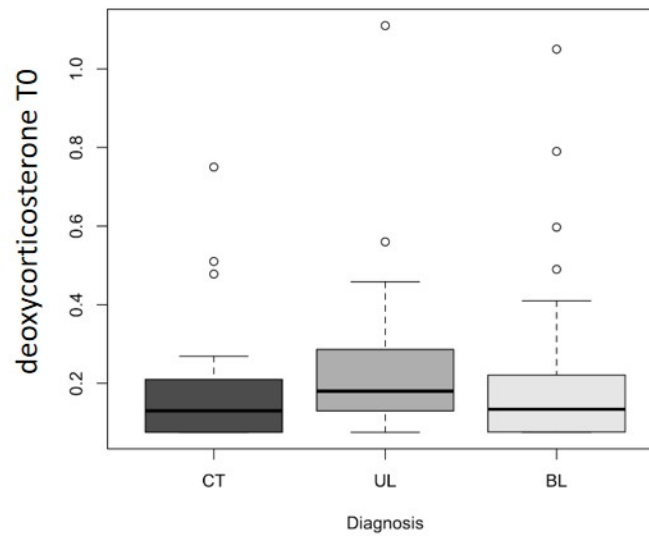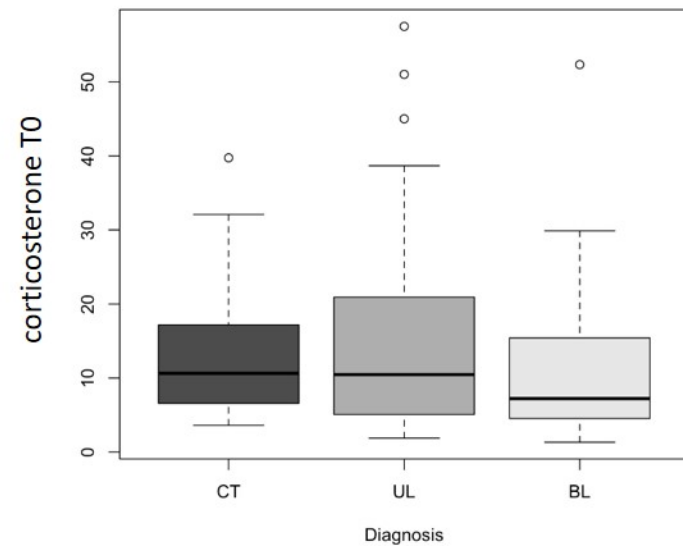

Supplement: Supplemental Figure 1: Comparison of progesterone, 17-hydroxyprogesterone, 11-deoxycortisol, deoxycorticosterone and corticosterone levels at basal state (T0) between CT subjects, UL patients and BL patients. Results are expressed in nmol/L. [file supplementary_figure_1.pdf]

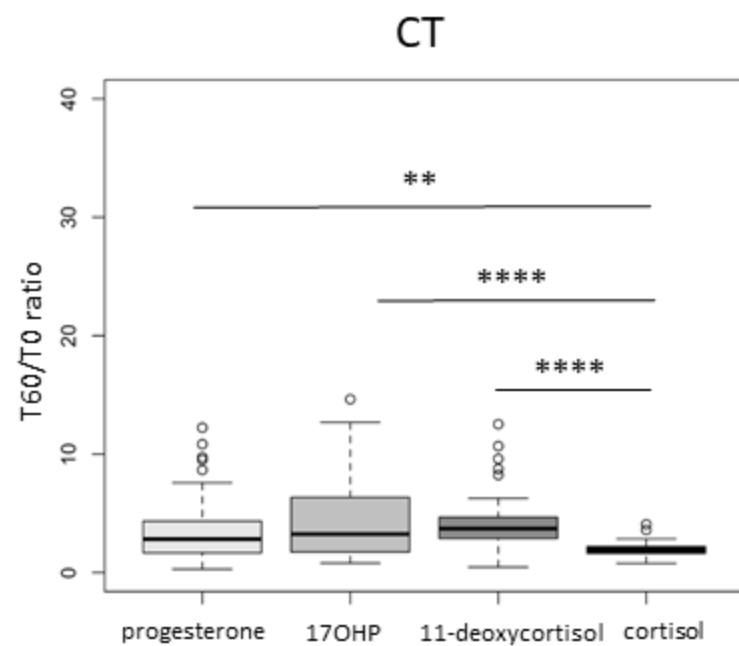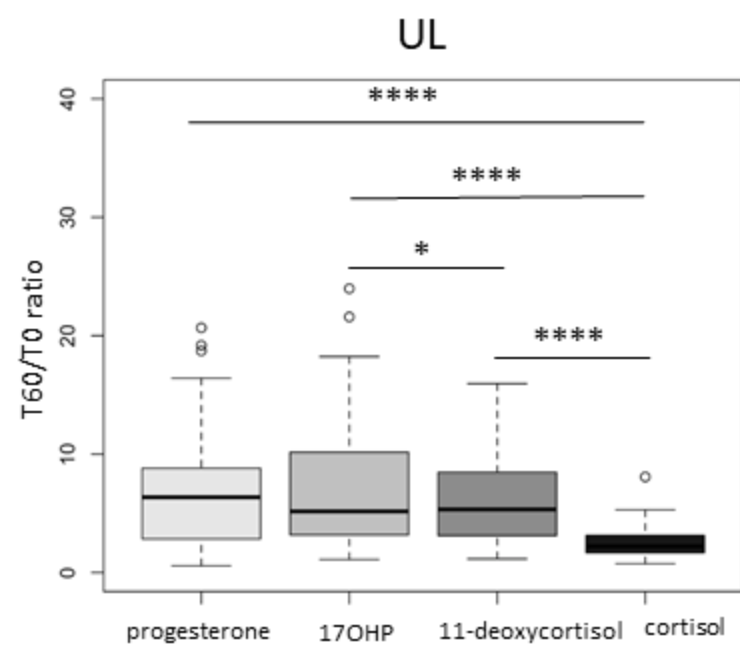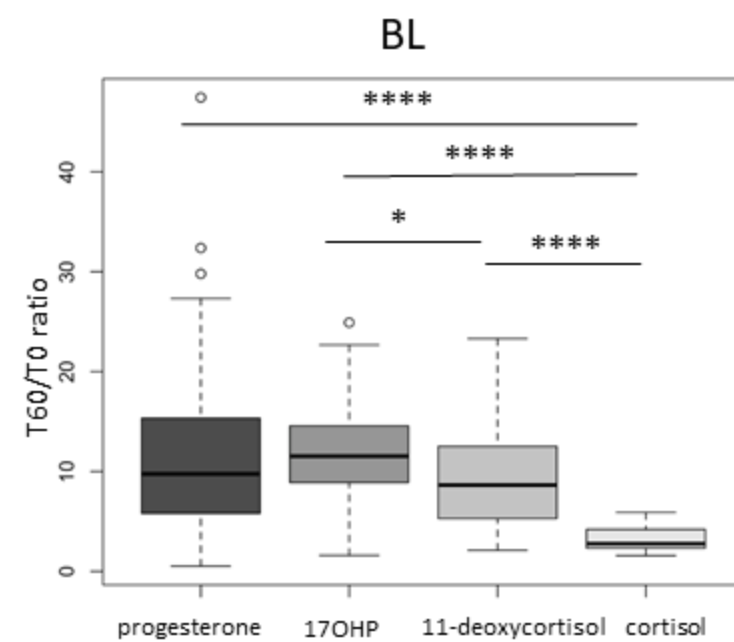

Supplement: Supplemental Figure 3: Decreasing amplitude of response to ACTH from downstream precursors to downstream bioactive cortisol on glucocorticoid pathways in the 3 groups : CT subjects, UL and BL patients. Amplitude of response to ACTH was evaluated by T60/T0 steroid concentration ratio. 17OHP: 17-hydro [file supplementary_figure_3.pdf]

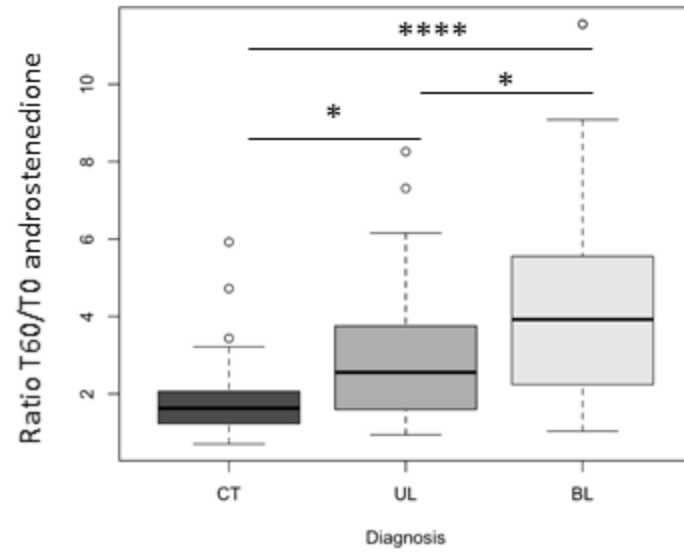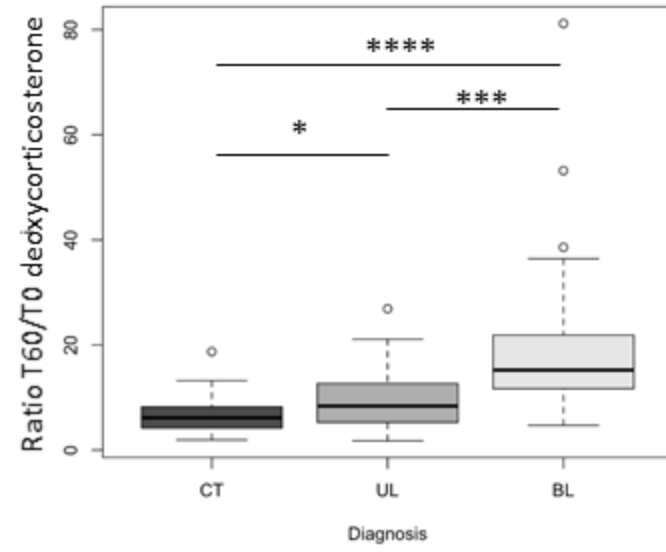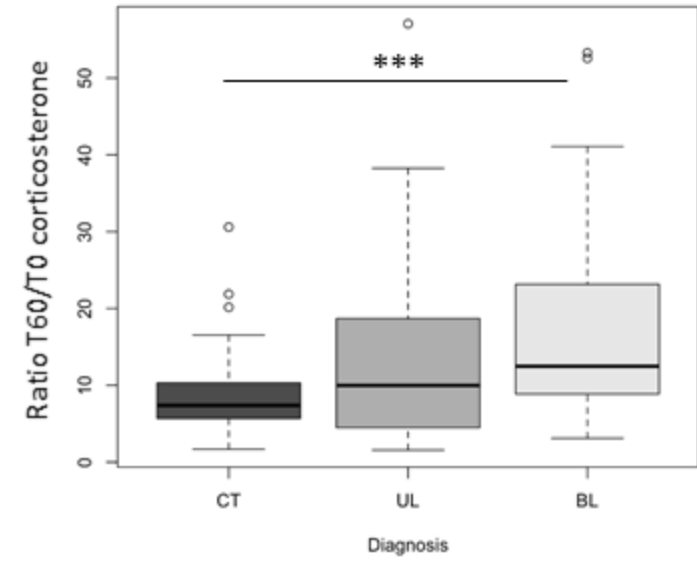

Supplement: Supplemental Figure 4: Comparison of the amplitude of response to ACTH of androstenedione and mineralocorticoids precursors (deoxycorticosterone and corticosterone) between CT subjects, UL patients and BL patients. The amplitude of response is higher in BL patients in comparison to both CT subjects  [file supplementary_figure_4.pdf]

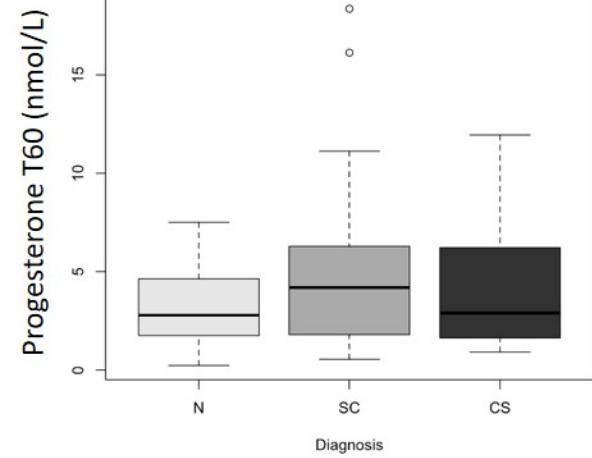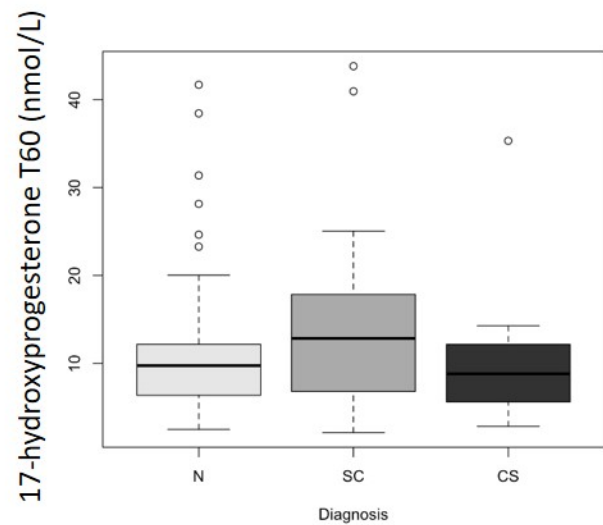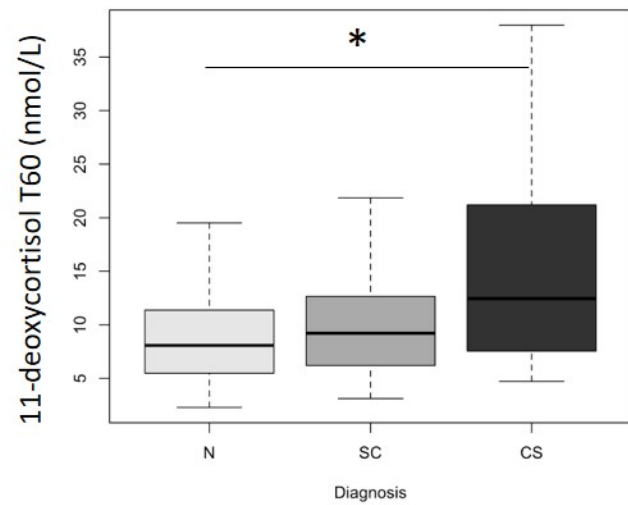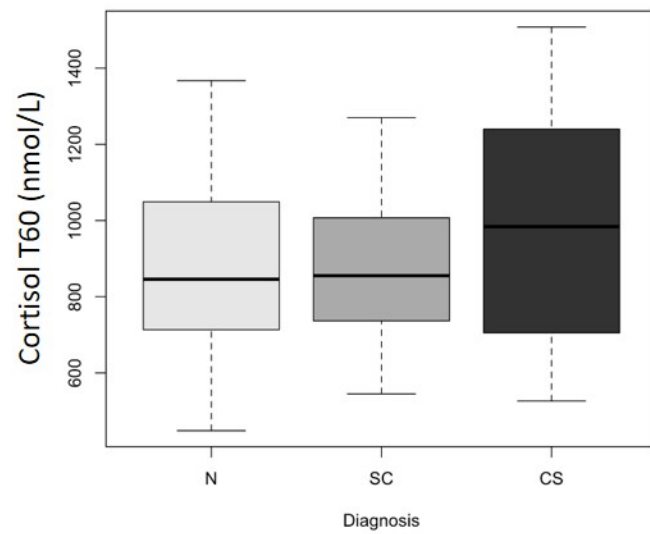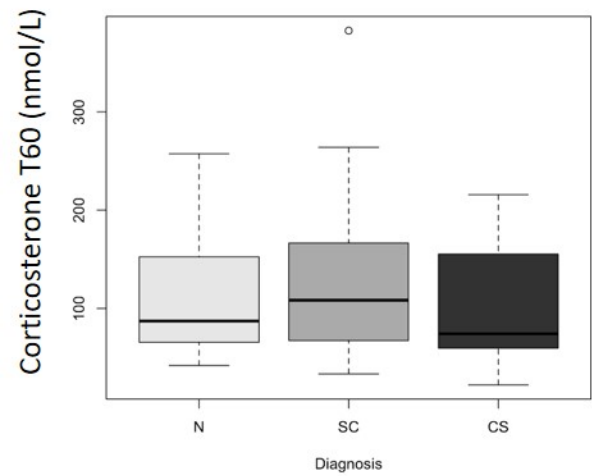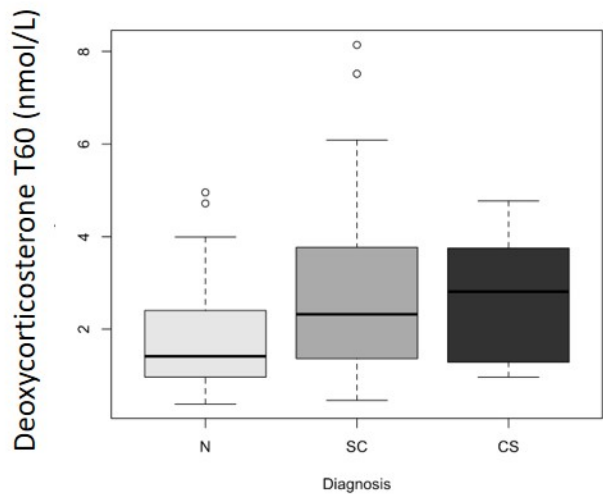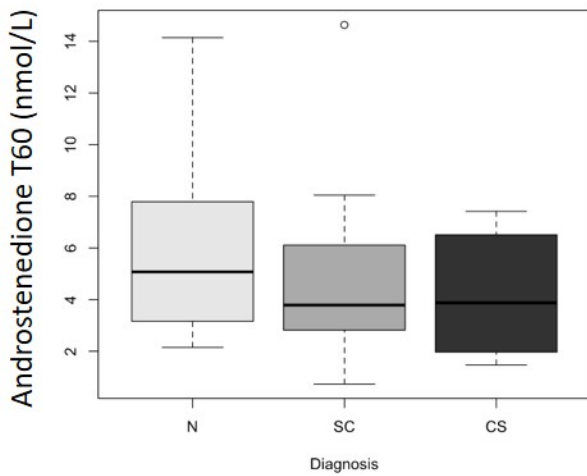

Supplement: Supplemental Figure 6: Comparison of progesterone, 17-hydroxyprogesterone, 11-deoxycortisol, cortisol, corticosterone, deoxycorticosterone, and androstenedione levels after ACTH1-24 stimulation (T60) between N patients, SC patients and CS patients (cortisol status level). Results are expressed in nm [file supplementary_figure_6.pdf]

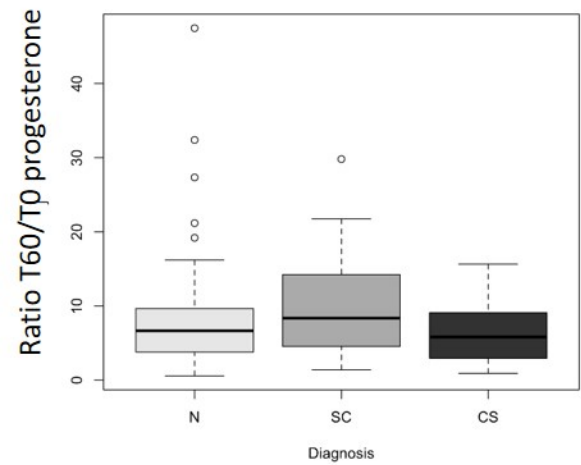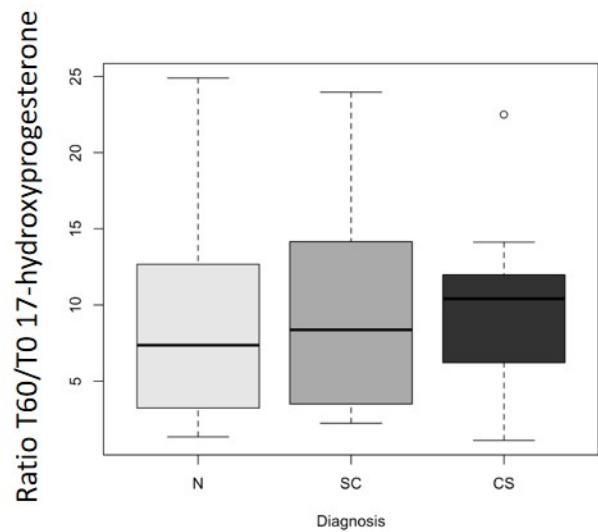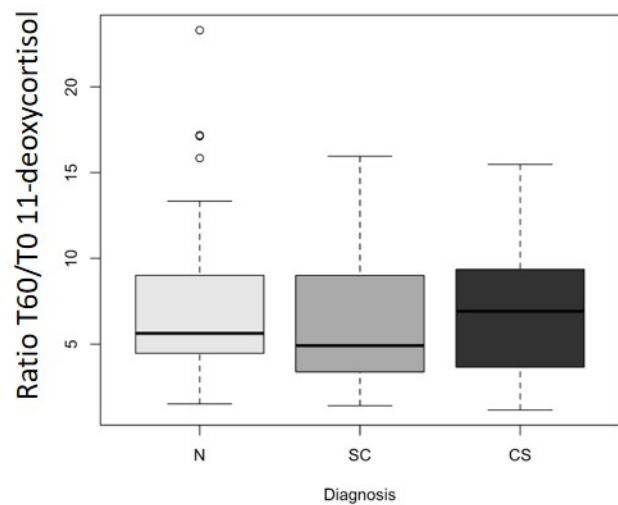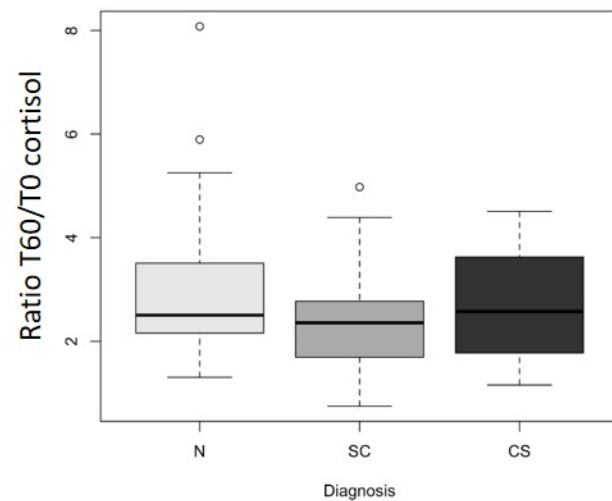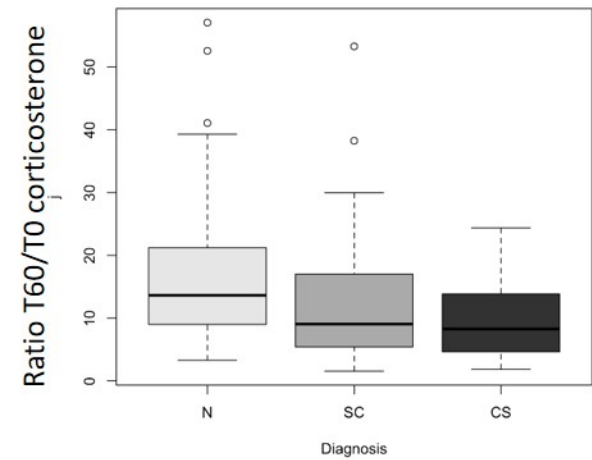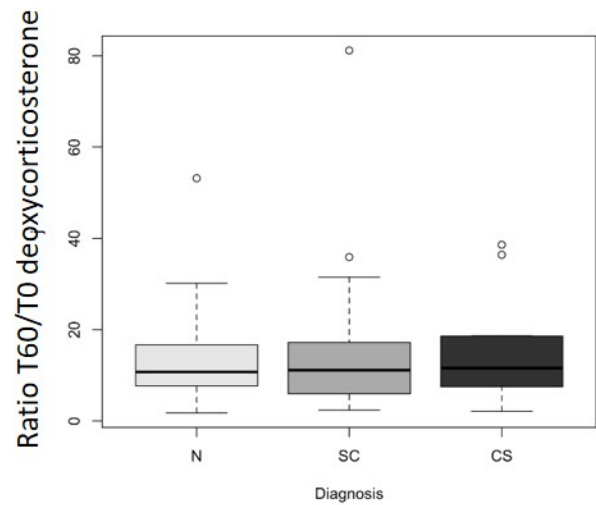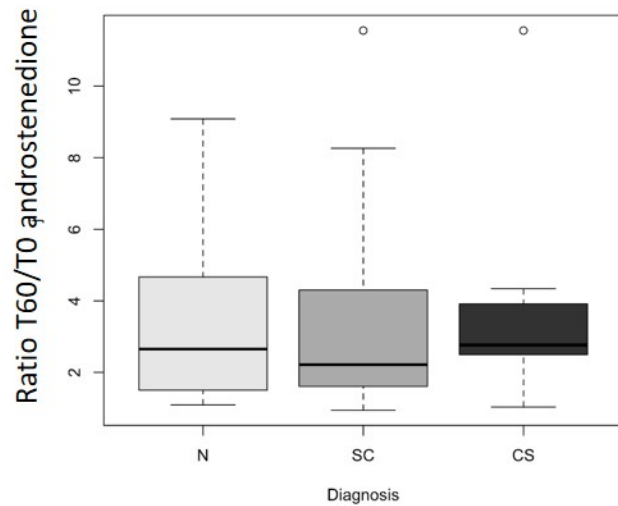

Supplement: Supplemental Figure 7: Comparison of the amplitude of response to ACTH of progesterone, 17-hydroxyprogesterone, 11-deoxycortisol, cortisol, corticosterone, deoxycorticosterone, and androstenedione between N patients, SC patients and CS patients (cortisol level status). [file supplementary_figure_7.pdf]

A

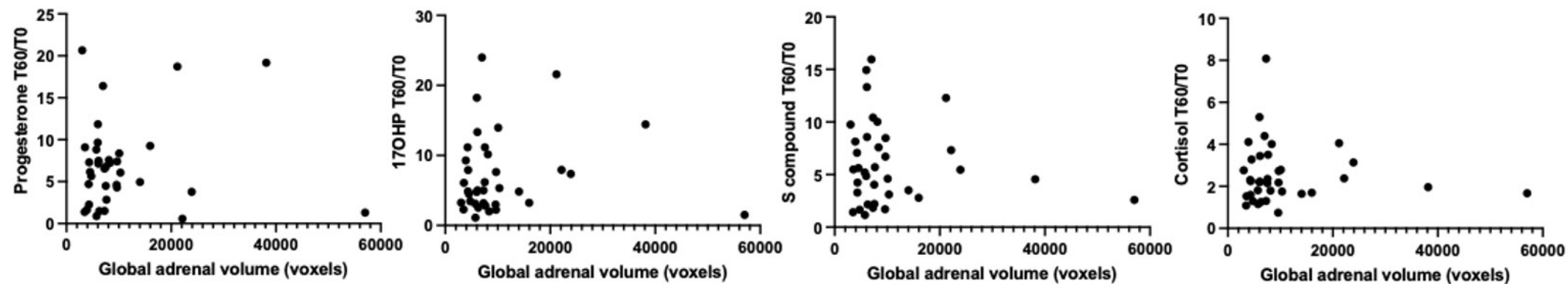

B

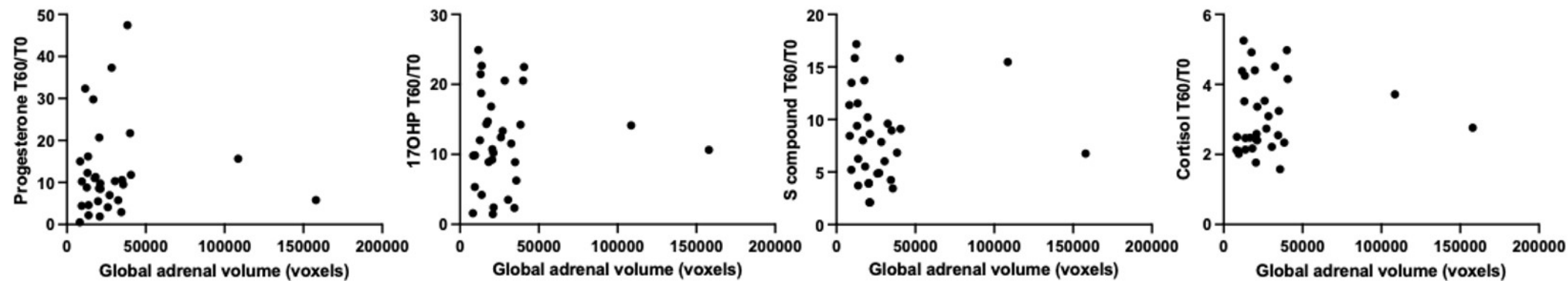

Supplement: Supplemental Figure 8: Relation between steroid amplitude of response to ACTH of glucocorticoids and precursors and adrenal volume. No correlation between any steroid amplitude of response to ACTH and adrenal volume was found in either UL (A) or BL patients (B). [file supplementary_figure_8.pdf]

### CYP11B1

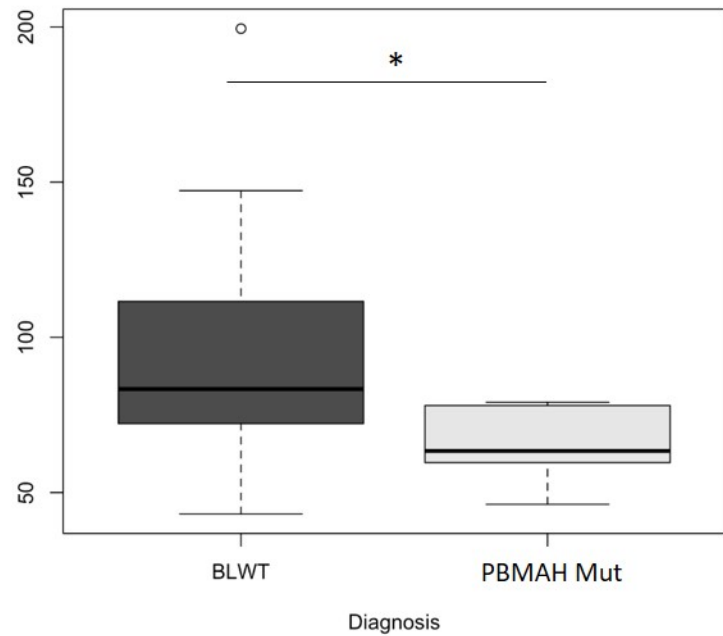

### corticosterone/deoxycorticosterone T60

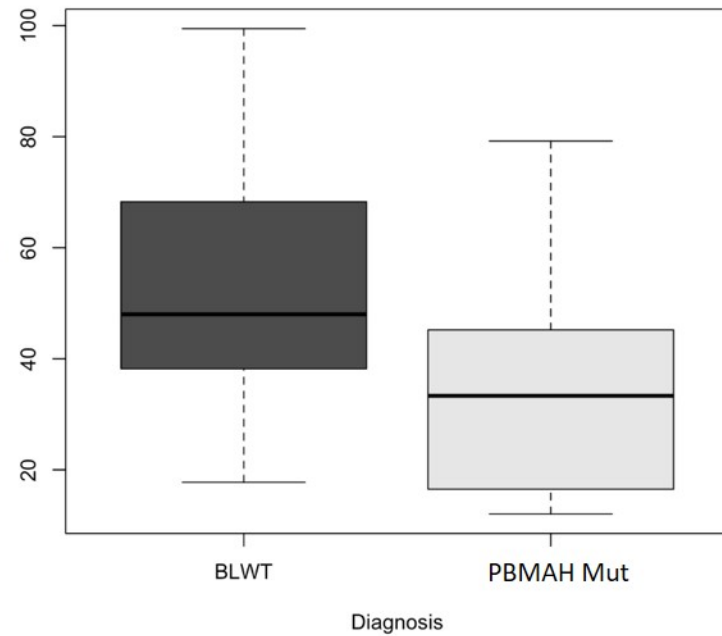

### 17OHP/progesterone T60

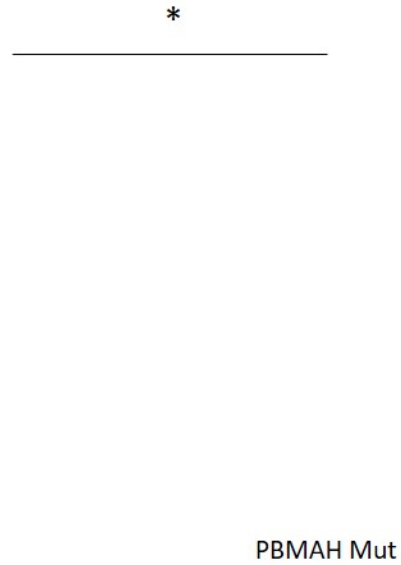

Supplement: Supplemental Figure 9: Enzymatic activity of downstream enzymes in BL patients according to the ARMC5 Genotype. CYP11B1 (glucocorticoids pathway), CYP11B2 (mineralocorticoids pathway) and CYP17A1 (androgens pathway) is decreased in PBMAH with ARMC5 mutation (PBMAH Mut) in comparison to BL patients w [file supplementary_figure_9.pdf]
