# Supplementary material for: Decreased steroidogenic enzyme activity in benign adrenocortical tumors is more pronounced in bilateral lesions as determined by steroid profiling in LC-MS/MS during ACTH stimulation test
Source: Endocr Connect. 2022 Jun 22;11(8):e220063. doi: 10.1530/EC-22-0063 (PMC9346343; doi:10.1530/EC-22-0063)
Supplement: Supplemental Figure 2: Comparison of the different steroids levels after ACTH1-24 stimulation (T60) between CT subjects, UL patients and BL patients. Results are expressed in nmol/L. * p< 0.05 ; ** p<0.001; *** p< 0.0001; **** p<0.00001. [file supplementary_figure_2.pdf]

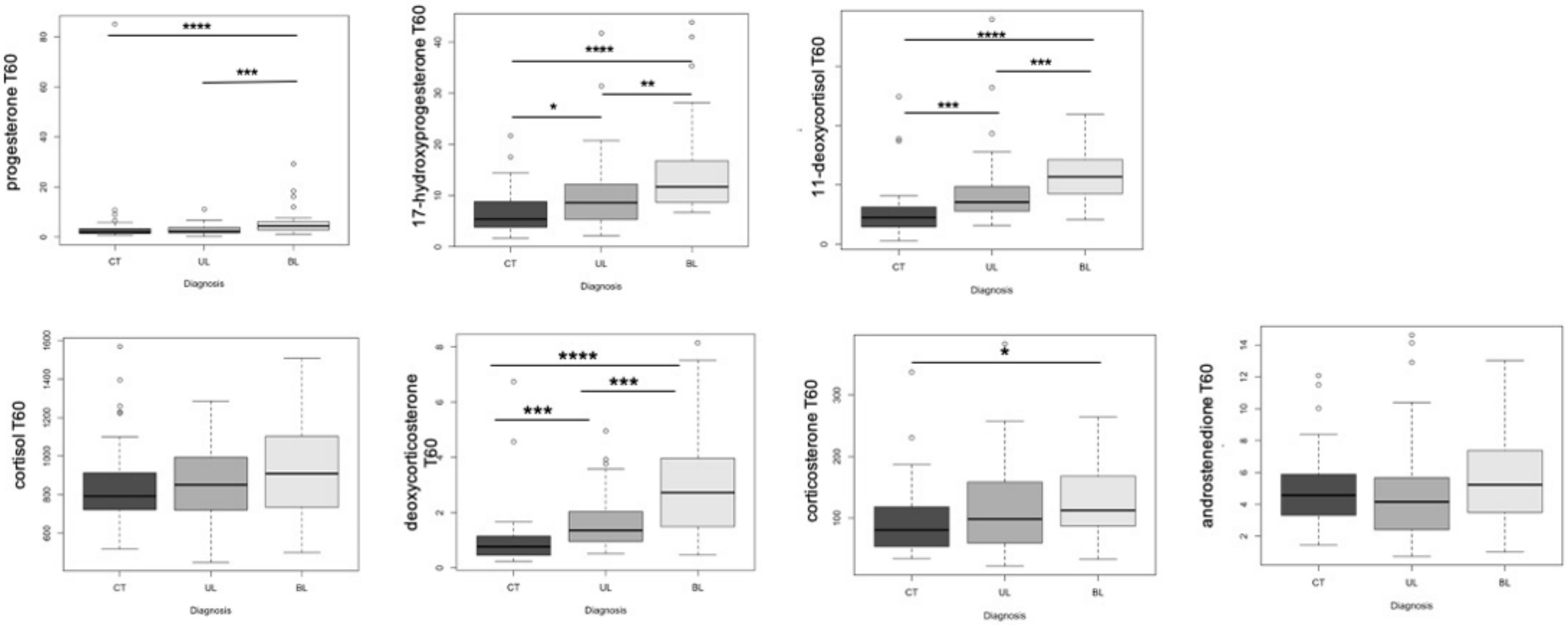

progesterone T60
17-hydroxyprogesterone T60
11-deoxycortisol T60
cortisol T60
deoxycorticosterone T60
corticosterone T60
androstenedione T60
CT
UL
BL
Diagnosis
****
***
**
*
