# Supplementary material for: Decreased steroidogenic enzyme activity in benign adrenocortical tumors is more pronounced in bilateral lesions as determined by steroid profiling in LC-MS/MS during ACTH stimulation test
Source: Endocr Connect. 2022 Jun 22;11(8):e220063. doi: 10.1530/EC-22-0063 (PMC9346343; doi:10.1530/EC-22-0063)
Supplement: Supplemental Figure 5: Comparison of progesterone, 17-hydroxyprogesterone, 11-deoxycortisol, cortisol, corticosterone, deoxycorticosterone, and androstenedione levels at basal state (T0) between N patients, SC patients and CS patients (cortisol status level). Results are expressed in nmol/L. * p< 0. [file supplementary_figure_5.pdf]

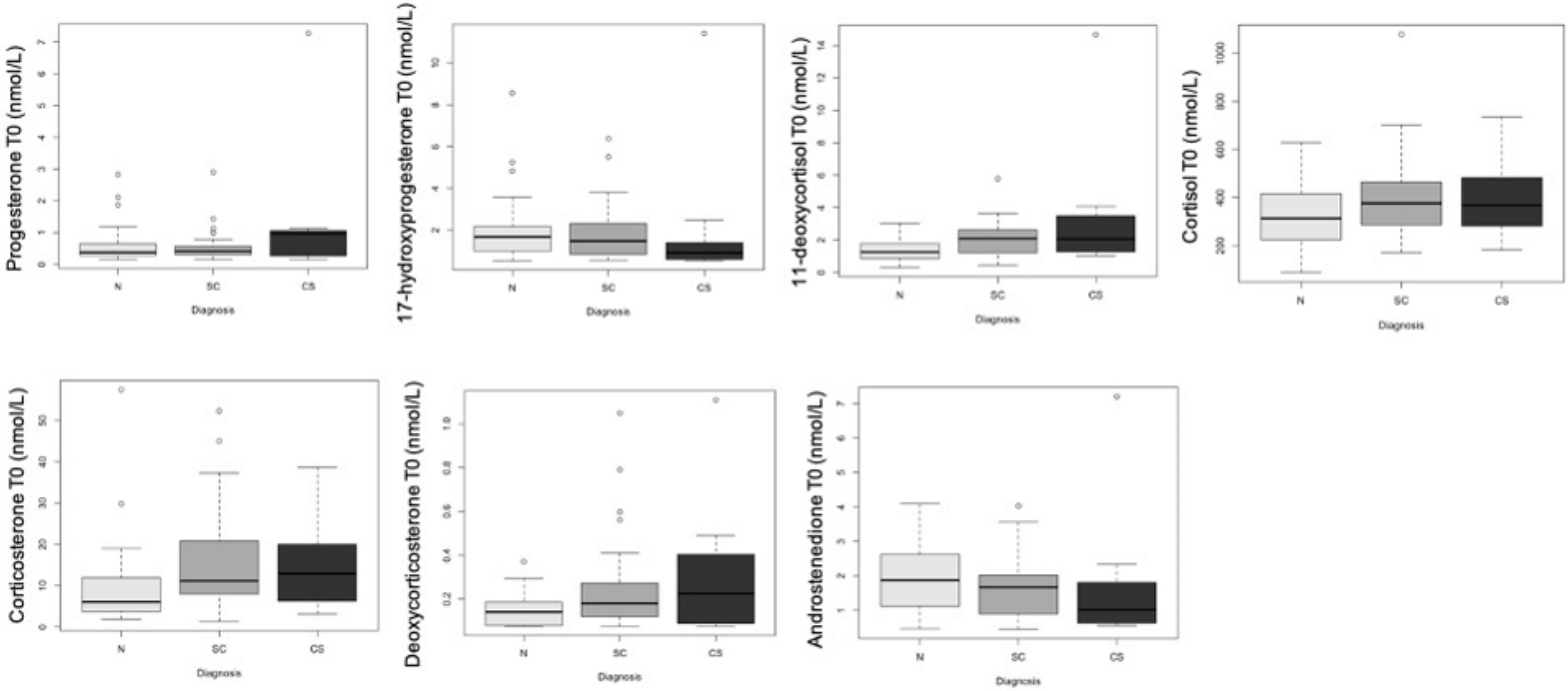
Progesterone T0 (nmol/L)
N
SC
CS
Diagnosis
17-hydroxyprogesterone T0 (nmol/L)
N
SC
CS
Diagnosis
11-deoxycortisol T0 (nmol/L)
N
SC
CS
Diagnosis
Cortisol T0 (nmol/L)
N
SC
CS
Diagnosis
Corticosterone T0 (nmol/L)
N
SC
CS
Diagnosis
Deoxycorticosterone T0 (nmol/L)
N
SC
CS
Diagnosis
Androstenedione T0 (nmol/L)
N
SC
CS
Diagnosis
