# Supplementary material for: Decreased steroidogenic enzyme activity in benign adrenocortical tumors is more pronounced in bilateral lesions as determined by steroid profiling in LC-MS/MS during ACTH stimulation test
Source: Endocr Connect. 2022 Jun 22;11(8):e220063. doi: 10.1530/EC-22-0063 (PMC9346343; doi:10.1530/EC-22-0063)
Supplement: Supplemental Table 1: Quantification and confirmation transitions, and retention time of each steroid molecule. [file supplementary_table_1.pdf]

**Steroid molecule**	**Quantification transition**	**Confirmation transition**	**Retention time (min)**
Cortisol-D4	*367.23 > 121.05*	*367.23 > 331*	*3.88*
Cortisol	363.21 > 121.07	363.21 > 327.2	3.90
Corticosterone-D8	*355 > 319*	*355 > 337*	*4.95*
Corticosterone	347 > 121	347 > 329	5.05
11-deoxycortisol	347.22 > 109.12	347.22 > 97.12	5.28
11-deoxycortisol-D5	*352.3 > 100.25*	*352.3 > 113.12*	*5.40*
Delta4-androstenedione	287.12 > 109.12	287.12 > 97.12	6.05
Delta4-androstenedione-C13	*290 > 112.12*	*290 > 100.13*	*6.06*
11-deoxycorticosterone-D8	*339 > 100*	*339 > 113*	*6.70*
11-deoxycorticosterone	331 > 97	331 > 109	6.90
17-hydroxyprogesterone-D8	*339 > 113.12*	*339 > 100.15*	*7.45*
17-hydroxyprogesterone	331.21 > 109.125	331.21 > 97.12	7.52
Progesterone-D9	*324 > 100.12*	*324 > 113.12*	*9.55*
Progesterone	315.29 > 97.12	315.29 > 109.12	9.55

Supplemental Table 1 Quantification and confirmation transitions, and retention time of each steroid molecule.
